# Harvesting krypton isotopes from the off-gas of an irradiated water target to generate ^76^Br and ^77^Br

**DOI:** 10.1038/s41598-022-05500-8

**Published:** 2022-01-26

**Authors:** Hannah K. Clause, Katharina A. Domnanich, Chloe Kleinfeldt, Morgan Kalman, Wesley Walker, Chirag Vyas, E. Paige Abel, Gregory W. Severin

**Affiliations:** 1 Department of Chemistry, Michigan State University, 578 S. Shaw Ln., East Lansing, MI USA; 2 Facility for Rare Isotope Beams, Michigan State University, 640 S. Shaw Ln., East Lansing, MI USA

**Keywords:** Nuclear chemistry, Imaging studies

## Abstract

A flowing-water target was irradiated with a 150 MeV/nucleon beam of ^78^Kr at the National Superconducting Cyclotron Laboratory to produce ^77^Kr and ^76^Kr. Real-time gamma-imaging measurements revealed the mass transport of the krypton radioisotopes through the target-water processing, or “isotope harvesting”, system. The production rates were determined to be 2.7(1) × 10^–4^ nuclei of ^76^Kr and 1.18(6) × 10^–2^ nuclei of ^77^Kr formed per incident ^78^Kr ion. Utilizing an off-gas processing line as part of the isotope harvesting system, a total of 7.2(1) MBq of ^76^Kr and 19.1(6) MBq of ^77^Kr were collected in cold traps. Through the decay, the daughter radionuclides ^76^Br and ^77^Br were generated and removed from the traps with an average efficiency of 77 ± 12%. Due to the differences in half-lives of ^76^Kr and ^77^Kr, it was possible to isolate a pure sample of ^76^Br with 99.9% radionuclidic purity. The successful collection of krypton radioisotopes to generate ^76^Br and ^77^Br demonstrates the feasibility of gas-phase isotope harvesting from irradiated accelerator cooling-water. Larger-scale collections are planned for collecting by-product radionuclides from the Facility for Rare Isotope Beams.

## Introduction

The upcoming Facility for Rare Isotope Beams (FRIB) at Michigan State University provides the scientific community with a vast array of radionuclides that can be utilized for a variety of different basic and applied science experiments^1^. As FRIB fulfills its mission of providing rare isotope beams for fundamental nuclear science research, many useful by-product radionuclides will be formed through beam interactions with accelerator components. Of particular interest are interactions between FRIB’s primary heavy-ion beam and a water-filled beam dump which will produce a wide variety of radionuclides. When the beam dump water is recirculated, it is possible to collect the radionuclides through a process termed “isotope harvesting”. Isotope harvesting has been identified as a source of otherwise-difficult-to-obtain radionuclides for use in basic and applied research^2^.

To test the proposed harvesting system to be used at FRIB, a similar system was designed and implemented in several beam experiments at the National Superconducting Cyclotron Laboratory (NSCL)^3^. This system was designed with the same materials on a smaller scale and was tested at lower beam power than planned for FRIB as well as with higher areal power deposition. These test experiments have included irradiation with several different stable isotope beams at different beam powers including ^1^H, ^40^Ca, ^48^Ca, and ^78^Kr^3–7^. The ^78^Kr irradiations are particularly interesting, because they provide an opportunity to simultaneously harvest radionuclides from the cooling water and its off-gas. The results from aqueous phase harvesting during ^78^Kr irradiation were discussed by Domnanich et al., while the current manuscript describes the gas-phase harvesting results^7^. For this work, a gas processing system was designed to move dissolved gaseous radionuclides that are produced in the water to gas collection traps. The motivation for the development was to capture ^76^Kr and ^77^Kr and generate medically relevant bromine radioisotopes, ^76^Br and ^77^Br, respectively.

Radioisotopes of bromine, such as ^76^Br and ^77^Br, are uniquely suitable radiolabels for small molecule theranostic radiopharmaceuticals. As a radiohalogen theranostic pair, ^76^Br and ^77^Br could become a valuable tool for systemic targeted radiotherapy^8–10^. Bromine-76 possesses many advantageous properties as a positron emission tomography (PET) imaging tracer, including a high percentage of positron emission (57%) and a long half-life (16.2 h), which enables the delivery of the radioisotope from the production site to point-of-use^8,9,11^. Bromine-77 (t_1/2_ = 57.0 h) is an Auger electron emitter which has promise for therapy in small disseminated tumors^10,12^. The advantages and disadvantages for the radiobromines in medical applications are thoroughly discussed in a recent review by Wilbur and Adam^13^.

Currently, the proton-rich radioisotopes of bromine are produced by proton irradiation of metal-selenides (e.g. CuSe or NiSe) which are enriched in ^76^Se or ^77^Se^14–16^. Very elegant techniques have been developed to optimize production but they have not been widely adopted for clinical radiopharmaceutical use. An alternative approach was pursued by De Jong and coworkers, which was based upon high-energy irradiation of bromide salts to create radiokryptons to generate the bromine isotopes^17^. They found that the formation of generators from the shorter lived ^76^Kr and ^77^Kr parents (14.8 h, and 1.24 h respectively), made it possible to isolate and use the radioactive daughters for different applications, including radiolabeling peptides and antibodies. Interestingly, the radiokryptons could also be used to radiobrominate small molecules through a process called excitation labeling^18^. More recently, McGuinness, Wilkinson and Peaslee investigated production of the radiobromides and radiokrypton parents by heavy-ion fusion-evaporation^19^.

The current work demonstrates the successful use of an isotope harvesting gas processing line to collect radioactive noble gases and generate pure radiohalogens. With extension to other noble gases, the Kr/Br work presented here has implications for future gas-phase harvesting at FRIB to create generators like ^122^Xe/^122^I or ^211^Rn/^211^At^1^.

## Experimental methods

### Irradiation and experimental set-up

A water-filled target, described by Domnanich et al^3^, was irradiated at the NSCL with 0–4 particle-nanoAmperes (pnA) of 150 MeV/nucleon ^78^Kr beam over the course of 11 h with a total integrated beam current of 16 pnA · h. Measurements of beam current from an unsuppressed target were recorded approximately every second; however, the values were calibrated against a Faraday cup which was inserted to intercept the beam at both low and high power periodically throughout the irradiation. The regularly recorded values from the unsuppressed target were corrected using a linear relationship between the accurate values measured with the Faraday cup and the relative measurements on the target. In addition to the periods of time where the Faraday cup intercepted the beam current, the beam was intercepted early in the beam line several times in order to collect water samples.

The irradiated water-filled target was installed at the end of an experimental beam line at the NSCL. The target was part of a flowing water system which included up to 36 L of water, circulated by a pump from a storage tank through multiple loops for different purposes. These loops included the main target loop which pumped water from the tank to the water-filled target and back, a peroxide decomposition loop to decrease the level of the potentially harmful radiolysis product, an aqueous chemistry loop where isotopes could be harvested from the water, and a gaseous chemistry line where isotopes could be harvested from the headspace described by Domnanich et al.^7^.

**Figure 1:**
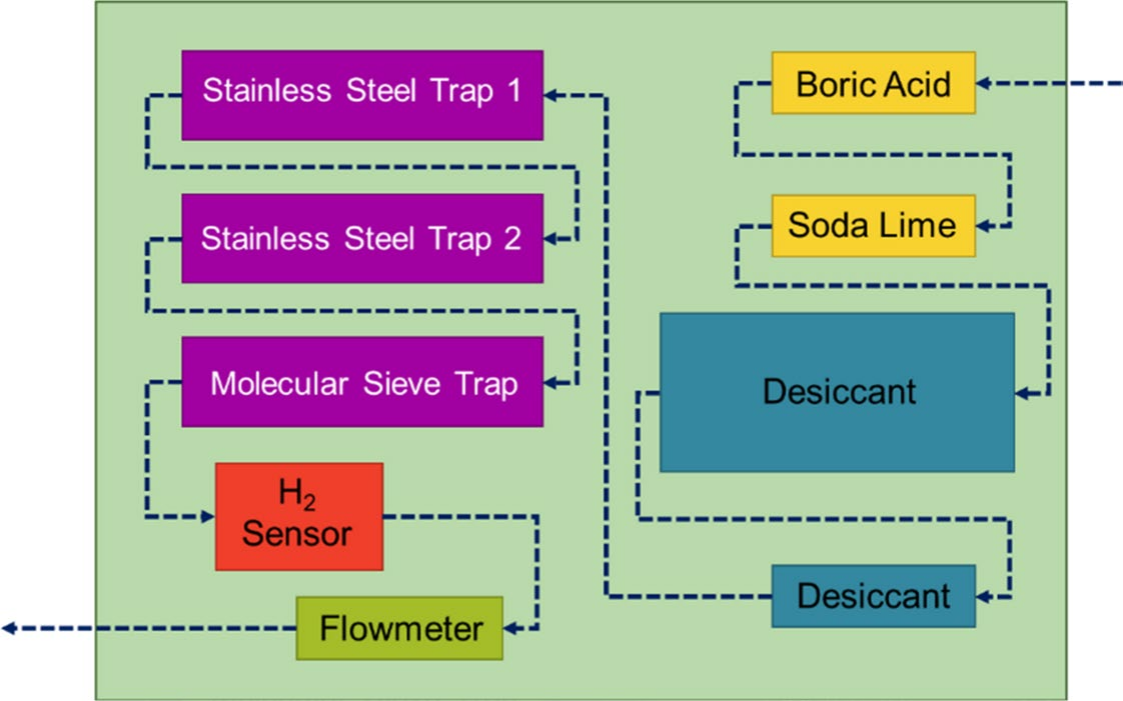
Schematic of the gas line representing the trapping components and measurement probes in the order the purge gas travelled through.

Using a helium purge-gas stream of 135 mL/min (Omega FMA5512A 0–500 mL/min Mass Flow Controller), gaseous radionuclides and radiolytically produced gases were sparged from the water into the headspace of the storage tank and carried downstream to the gaseous chemistry line. The gaseous chemistry line consisted of a series of gas traps to purify the gas stream and remove water vapor and from the mixture to optimize the collection of gaseous krypton isotopes. A schematic of the gas line is shown in Fig. 1. The first of these traps was a column filled with boric acid (Fisher Chemical, crystalline, certified ACS), which is known to capture ammonia^20^ – a gaseous species which could be produced by reactions between radiogenically produced ^13^ N and radiolytically produced H_2_ in the water during irradiation. Next in the series was another column filled with soda lime (Sigma Aldrich granular, ACS reagent, + 100 mesh), which is a highly efficient absorber for any carbon dioxide present as [^11^C]CO_2_^21^. Both of the first two columns were made from 14 cm of polypropylene tubing (McMaster Carr, OD ¾”, 19 mm) with glass wool packing on both ends to contain the chemicals. Following the first two traps was a large cannister (McMaster Carr, polypropylene filter housing for 10″ (25.4 cm) cartridges part no. 44195K11 with a polypropylene 9 ¾” (25 cm) filter cartridge part no. 9007T51) filled with desiccant (Drierite, indicating, 8 mesh) and a smaller clear column filled with more desiccant to allow visual assessment of any color change in the indicator.

The next segment of the gas line included a series of cold traps, used to trap krypton online. First in the series were two stainless steel traps each made with a fritted stainless-steel filter, featuring a large, porous surface (Swagelock, SS-6F-60, 316 stainless steel, 60 micron pore size) and approximately 60 cm of stainless steel tubing (316 stainless steel, ¼” OD, 6 mm, McMaster-Carr). Both traps were placed in a dewar filled with liquid nitrogen for cooling. Following the stainless-steel cold traps was a gas washing bottle trap, filled with molecular sieves (Sigma Aldrich, pellets, 4 Å) also placed in a dewar filled with liquid nitrogen. *Safety note: it should be noted that upon warming the traps pressurize due to desorption of gases from the molecular sieves. Therefore, care must be taken to not overpressurize the fittings and housings in the event of loss of cryogenics, or when allowing the traps to warm.

Following the traps was a hydrogen analyzer (Hy-Optima 700B Series In-Line Hydrogen Process Analyzer). During the irradiation of water, molecular hydrogen is radiolytically produced. By measuring the percentage of hydrogen in the gas stream, predictions of the mass transport of the other gases through the system could be made. Another important sensor in the gas line was a floating-ball flowmeter (Brooks 0 to 400 ccm Variable Area Mechanical Flowmeter), used to confirm a consistent flow rate of the gas at the end of the line after flowing across all traps. This was important to ensure that no obstructions were forming in the traps and all gas that was flowing into the gas line was also flowing out. *Safety note: H_*2*_* can build up and pose an explosion hazard. Therefore, gas flow rates within the line were selected to ensure that the H*_*2*_* content in the components would not reach the lower explosive limit in air (4%) if released.*

The final segment of the gas line contained a delay line of approximately 150 m of polypropylene tubing (OD ¼”, 6 mm, McMaster Carr), leading to two air-tight collection bags (Restek Tedler 100 L Sampling Bags) to collect all gases that were not trapped online during the irradiation.

### Radionuclide detection and quantification

During the irradiation, a PHDS Nuclear Physics Imager (NPI) (PHDS Inc, Knoxville TN, USA) was used to measure the real-time distribution of activity through the harvesting system. This portable detector has the capability to detect, identify, and spatially quantify radioactivity by high-resolution, gamma-ray spectroscopy using a pinhole collimator and a position sensitive, segmented, high purity germanium crystal. During this experiment, NPI pinhole images were used to identify, locate, and quantify ^77^Kr using the gamma energy of 129 keV. The detector was placed approximately 1.7 m away from the aqueous and gas chemistry boxes. In this configuration, the spatial resolution was approximately 3 cm. In order to reduce neutron fluence on the detector, three 5-gal water-filled carboys were used as shielding.

**Figure 2:**
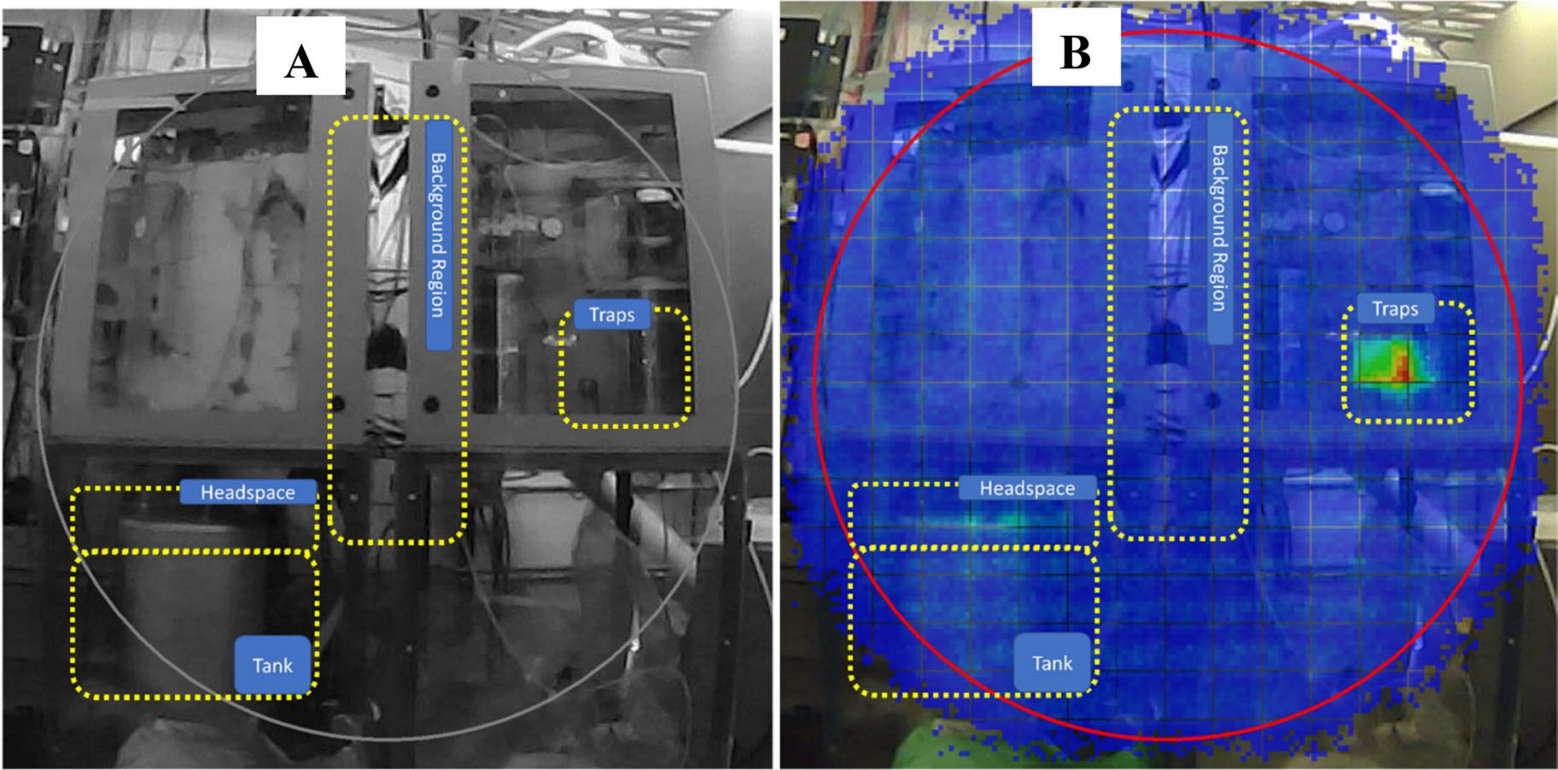
Example of the images collected with the NPI detector of the (**A**) experimental set up and a (**B**) heat map of the radiation produced and collected in the system. The ROIs selected to determine counts of activity in the headspace and the tank, the traps, and background measurements are depicted by the yellow boxes.

Pinhole collimated gamma spectra were collected with the NPI at different time points during beam-off periods (The interaction of neutrons with the detector impeded the collection of data while the beam was directed on-target). With the Imager 32 Software (PHDS Inc) dead-time corrected net photopeak count rates at 129 keV were integrated over regions of interest (ROIs): the water tank (including its headspace), the cold traps, and a background region between the collection boxes. The locations of the ROIs are shown in Fig. 2A, overlaid on the photograph of the experimental set-up. A representative heatmap showing the pinhole collimated 129 keV signal intensity in the field of view is shown in Fig. 2B. These measurements in-between irradiation periods were used to determine the movement of ^77^Kr through the system and were coupled to a mass transport model to determine the ^76/77^Kr isotope production rates.

For comparison to the NPI data, the mass transport of ^76/77^Kr through the harvesting system was modeled with a set of first order differential equations. Those equations are included in the Supplemental Materials.

After the irradiation, the boric acid and soda lime traps, the three cold gas traps, and the two air-tight gas collection bags were removed from the system and counted with a Canberra Broad Energy Ge Gamma-ray Detector (BE2022). The detector had been calibrated using a sealed ^152^Eu source, and additional calibrations were made by placing the ^152^Eu source inside one of the cold-trap dewars to accurately account for attenuation through the material. The Genie 2000 Software (Mirion Technologies) was used to analyze the data.

### Trapping and collection of krypton isotopes

Using the gas line described above, gaseous isotopes of krypton (^76^Kr and ^77^Kr) were trapped on the cold molecular sieve trap and cold stainless-steel traps during irradiation and for another four hours after irradiation was stopped.

To allow safe removal and separation of the traps, all traps were plumbed with valved quick-release fittings (Colder NS4 series couplings, ¼” NPT) As an additional precaution, the stainless-steel cold traps contained Swagelock turn valves (316 stainless steel sealed valve, ¼” Swagelock tube fittings) as well, which were closed before unhooking the quick-release valves. In order to quantify the amount of ^76/77^Kr gas which was trapped in the collection vessels, gamma-ray spectroscopy was performed on each of the traps approximately two hours after removing them from the gas line.

### Generation of bromine isotopes

**Figure 3:**
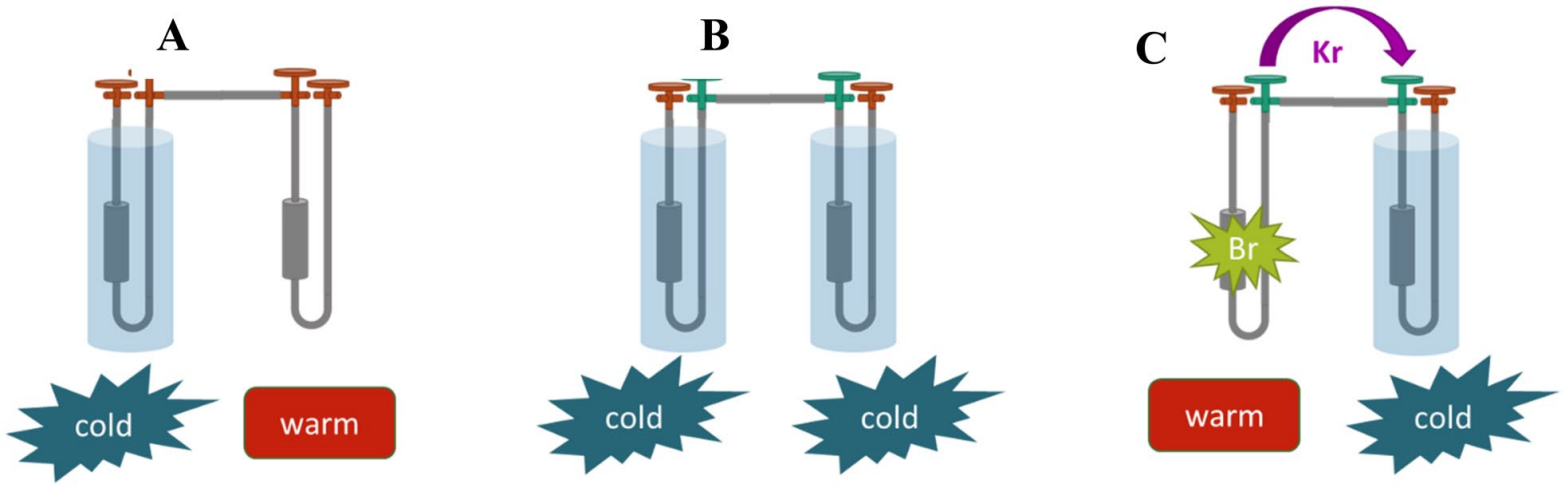
(**A**) The initial trap is cold, and the receiving trap is warm. (**B**) Both traps are cold and the valves between them are opened to allow gas transfer. (**C**) The initial trap is warmed, forcing gas into the receiving trap. In this process, the daughter ^76/77^Br isotopes would remain in the initial trap and the parent ^76/77^Kr isotopes would transfer to the receiving trap.

Following the post-irradiation gamma-ray measurements of the molecular sieve trap, both stainless steel traps, and each gas collection bag, an offline experimental set-up was developed to test the generation of ^76^Br and ^77^Br. All operations were carried out in a ventilated fume hood which was approved for radiological work. This was performed by connecting two traps using valves and polypropylene tubing and cryogenically transferring the collected gases from one trap to another. A schematic of this process is shown in Fig. 3.

**Figure 4:**
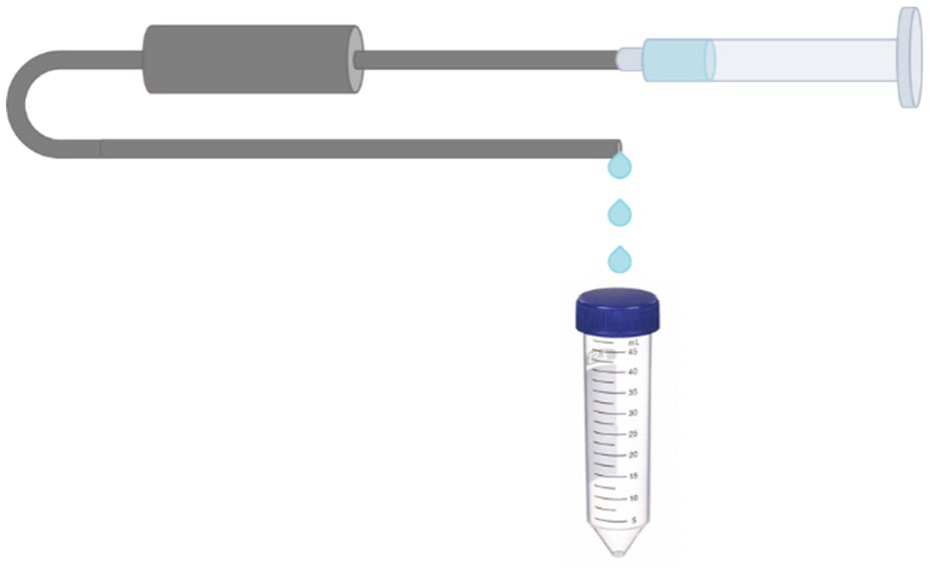
Example of the elution wash process of attaching a syringe of water to each trap and passing varying amounts of water through to a collection vessel.

An initial transfer was used to combine all the collected ^76/77^Kr gas from the molecular sieve trap and the first stainless-steel trap (trap 1) onto the second stainless steel trap (trap 2), using the process shown in Fig. 4. After allowing approximately 6 h residence time in trap 2 for ^76/77^Kr isotopes to decay into ^76/77^Br, the krypton was transferred to another stainless-steel trap (trap 3). At that time all captured ^77^Kr had decayed, leaving only ^76^Kr as a parent. After approximately 28 h of decay, the small amount of leftover ^76^Kr was transferred out of trap 3 into a discard trap. In all transfers, the ^76/77^Br isotopes did not move, leaving traps 1 and 2 with mixed samples of ^76^Br and ^77^Br, while trap 3 held purified ^76^Br.

### Elution of generated radiobromines

In order to elute the trapped bromine which remained in the stainless-steel trap post-transfer, an experimental set-up was designed to flow water through each trap and to collect the eluent into individual sample vials. This was done by removing the quick release valves and Swagelok turn valves from the inlet and outlet of each trap. At the entrance of each trap, the valves were replaced by a syringe in order to dispense specific volumes of water through the trap to be collected on the other end. A schematic of this set-up can be seen in Fig. 4.

The activity of ^76/77^Br in each trap, 1, 2, and 3, was assessed by gamma-ray spectroscopy before and after elution. The volumes of water used for each eluting each trap can be found in Table 1. There were two separate elutions of stainless-steel traps 1 and 2 and three elutions of trap 3. The percentage of the total ^76/77^Br activity removed from each trap was calculated.

**Table 1:** Volumes of water used to elute ^76/77^Br isotopes from traps 1, 2, and 3.

Trap Name	Elution 1 Volume (mL)	Elution 2 Volume (mL)	Elution 3 Volume (mL)
trap 1	17	10	–
trap 2	10	10	–
trap 3	7	4	10

## Results and discussion

### Radionuclide production

Using the NPI, 13 position-sensitive gamma-ray measurements were made throughout the irradiation. The results and respective uncertainties are represented in Fig. 6a and b and are provided in tabular form in the Supplemental Material. By fitting the mass-transport predictions to the NPI data, the rate constant for ^77^Kr transfer from the liquid phase to the gas phase, (defined as *k*_*1*_ in the Supplemental Materials), was found to be 4.1(4) × 10^–5^ Hz. The inefficiency of trapping, δ, was consistent with zero within an uncertainty of 4 × 10^–8^ Hz. Once the krypton-specific constants were determined, it was possible to benchmark the NPI measurements against the calibrated, quantitative, BEGe gamma-ray spectra to find the production rates for ^76^Kr and ^77^Kr: 2.7(1) × 10^–4^ nuclei of ^76^Kr, and 1.18(6) × 10^–2^ nuclei of ^77^Kr were formed per incident ^78^Kr ion.

**Figure 5:**
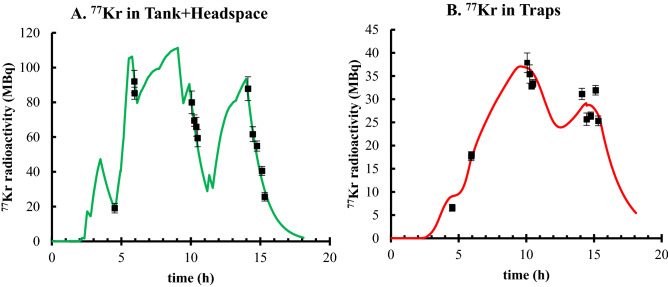
The mass transport of ^77^Kr was predicted for different locations within the harvesting system – (**A**) the water and the headspace in the tank (green) and (**B**) the traps (red). Real time data of the ^77^Kr activity calculated from the gamma spectra from the NPI detector are shown for comparison to the predictions.

**Figure 6:**
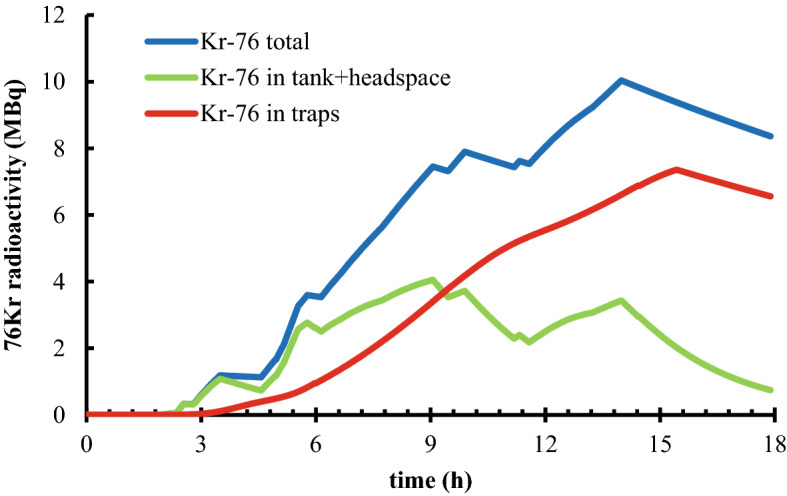
The predicted mass transport of ^76^Kr for different locations within the harvesting system – (blue) the total ^76^Kr, (green) the ^76^Kr in the tank and the headspace of the tank, (red) the ^76^Kr in collected on the traps.

The complex time structures in Fig. 5A and B result from the intermittency of beam-on and beam-off periods that were necessary for beam tuning and system checks. The NPI data matched the time structure, which was essential for determining the production rates of each ^76/77^Kr isotope. The large number of gamma emitters present in the system made it difficult to window on any other gamma ray than the 129 keV gamma-ray from ^77^Kr decay^7^. However, by determining the mass-transport behavior of krypton via ^77^Kr mapping, it was possible to calculate the time structure for ^76^Kr transport, shown in Fig. 6 in the absence of direct measurements. Around the 16-h time point in the graphs, the traps were removed from the harvesting system and the gas flow was diverted to the gas capture bags (causing the abrupt change in slope for the ^*76*^*Kr in traps* curve). At that time there was residual ^76^Kr in the tank and headspace which could have been recovered with prolonged trapping. In the future, the informed mass-transport model can be used to optimize the time at which the traps are removed from the system for processing.

The traps were removed from the system in order to measure the amount of krypton gas which was collected. A total of 7.2(1) MBq of ^76^Kr and 19.1(6) MBq of ^77^Kr were trapped in the online collection system as shown in Table 2. The table demonstrates how much activity was contained in each trap, with the molecular sieves trapping the most gas. Although Kr is generally retained on liquid nitrogen cooled stainless-steel, it is likely that the perpetual heat load of the incoming carrier gas promoted Kr mobility through traps 1 and 2 resulting in low Kr retention there. Neither the boric acid nor the soda lime traps were radioactive after the experiment, indicating that volatile ^7x^Br species, such as [^7x^Br]HBr, did not readily evolve from the water. Largely, Br isotopes which were produced directly from the beam or by decay of dissolved Kr isotopes accumulated on the anion exchange resins and catalytic converter^7^.

**Table 2:** Production and the location of online collection results of ^76^Kr and ^77^Kr*.

Trap Name	Activity of ^76^Kr (MBq)	Activity of ^77^Kr (MBq)
Trap 1	0.089(5)	0.48(2)
Trap 2	0.51(1)	2.05(8)
Molecular Sieve Trap	6.6(1)	16.6(6)
Total Activity	7.2(1)	19.1(6)

*All activity was measured after the irradiation at approximately 16 h in relationship to the plots in Figs. 5 and 6.

### Generation of bromine isotopes

^76^Br was isolated from ^77^Kr, ^76^Kr, and ^77^Br through a series of transfers. The first transfer, which involved combining the gaseous krypton isotopes that were collected online during irradiation, is qualitatively demonstrated with the BEGe gamma-ray spectra, shown in Figure S1. A small portion of the ^76/77^Kr gas remained in the molecular sieve trap after the transfer, but overall, 0.99(1) MBq of ^76^Kr and 3.8(2) MBq of ^77^Kr were transferred onto trap 2.

**Figure 7:**
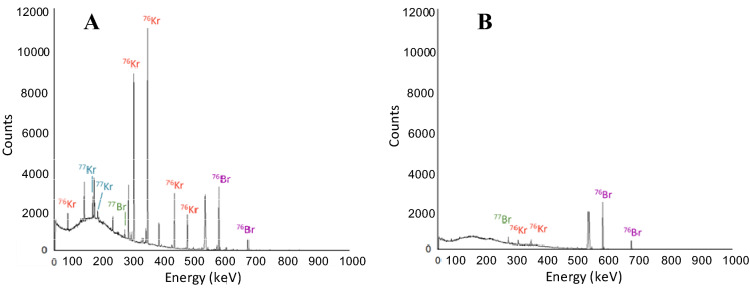
Gamma spectroscopy measurements (count time = 300 s) of stainless-steel trap 2 before (**A**) and after (**B**) the transfer of krypton gas to stainless steel trap 3. The successful movement of krypton gas due to changes in temperature is demonstrated by the decrease in counts of activity of ^76^Kr and ^77^Kr.

Following the accumulation of all gaseous activity onto trap 2, the ^76/77^Kr isotopes were allowed to decay for approximately 7 h. Then the activity of trap 2 two was re-measured before the transfer, and the gamma measurement results can be seen in Fig. 7A. The activity of ^77^Kr had decreased significantly from 3.8 to 0.03 MBq, making it possible to prepare a sample of ^76^Br without ^77^Br as a contaminant. The transfer from stainless steel trap 2 to stainless steel trap 3 resulted in some ^76^Kr remaining in trap 2 along with ^76^Br and ^77^Br, as shown in Fig. 7B and detailed in Table 3. The decay of ^76^Kr immobilized in stainless steel trap 3 generated pure ^76^Br (Figure S2).

**Table 3:** The decay-corrected activity measurements of krypton pre and post transfer from trap 2 to trap 3.

	Activity Pre-Transfer (MBq)		Activity Post-Transfer (MBq)	
	^76^Kr	^77^Kr	^76^Kr	^77^Kr
Trap 2	0.632(4)	0.030(1)	0.052(2)	
Trap 3			0.470(2)	0.0148(2)

**Figure 8:**
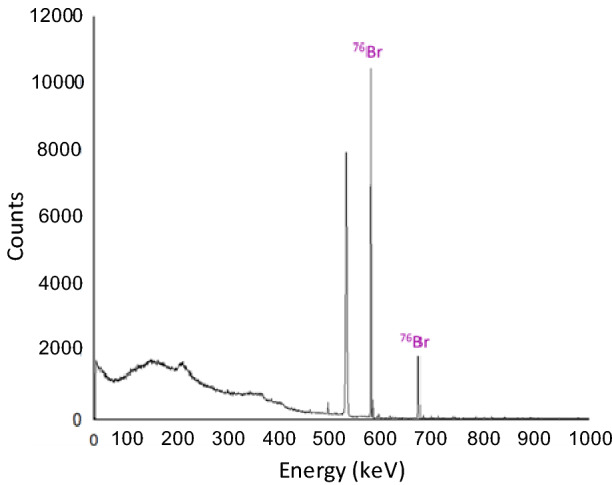
Gamma spectroscopy measurements (count time = 300 s) of stainless steel trap 3 after the transfer of krypton-76 gas to stainless steel trap 4. The successful movement of krypton gas due to changes in temperature is demonstrated by the lack of counts of activity of ^76^Kr. The isolation of ^76^Br is demonstrated.

The final transfer from stainless steel trap 3 to stainless steel trap 4 left pure ^76^Br in trap 3 and removed the ^76^Kr gas that still remained, which is demonstrated by the absence of any contaminating gamma rays in the gamma spectrum in Fig. 8 and Figure S3. Through the transfer process, it was demonstrated that ^76^Br could be produced and isolated from ^77^Br due to the differences in half-lives of their parent isotopes. The radionuclidic purity of the ^76^Br was 99.9%.

### Elution of generated radiobromines

Using the previously described elution procedure, the bromine remaining in each trap after the transfer of krypton, was eluted using different volumes of water. Performing quantitative gamma spectrometry measurements before the rinsing steps and of each eluent vial, the efficiencies of the procedure were determined. An example of the differences in gamma spectrometry measurements before, during, and after the rinsing procedure can be seen in Figure S4.

The rinsing procedure of trap 1, which was directly removed from the online set up, consisted of two separate elutions with 17 mL and 10 mL water, respectively. This was the first trial of the rinsing procedure, and as such, a larger volume of water was used to test the ability to remove the ^76/77^Br from the trap with water. In total, 89% of the ^76/77^Br contained in the trap was eluted with the 27 mL of water. The rinsing of trap 2 was also performed in two consecutive steps, while the water volume was reduced to 10 mL, facilitating a total elution efficiency of 79%. And finally, the rinsing procedure of trap 3 consisted of three separate elutions with 7 mL, 4 mL, and 10 mL of water, respectively. In this rinsing trial, only ^76^Br was present in the trap to be removed, and the elution efficiency was 64%.

The elution efficiency of ^76/77^Br decreased with decreasing total volume (Table S2) and had the lowest efficiency in the trial using a greater number of small volume washes. While it is important to use small volumes of water for eventually using the eluted bromine in radiolabeling procedures, it is also important to obtain as much bromine from the traps as possible to increase overall production yields using this method. Overall, given the high solubility of bromine in water, the elution efficiency was lower than expected, and this could be due to incomplete wetting of the generator surfaces. Additionally, speciation could play a role in the inefficiency. De Jong, Brinkman and Van Halteren observed formation of organic and other unidentifiable bromine species when eluting *in vacuo* produced radiobromines from generators. ^22^ They found that adding hydrogen containing gases to the krypton environment improved extraction of bromine isotopes and led to near-uniform speciation as bromide. In future experiments, similar approaches will be considered.

## Conclusions

The irradiation of a novel beam blocker by a stable beam of 150 MeV/nucleon ^78^Kr at an average beam current of 1.9 pnA for approximately 11 h facilitated the successful collection of 7.2(1) MBq of ^76^Kr and 19.1(6) MBq of ^77^Kr. This irradiation experiment utilized a harvesting system which has previously demonstrated the ability to collect radionuclides of interest from the aqueous phase; however, in this case, radionuclides were simultaneously harvested from the water and the gas line to be used as offline generators.

During this irradiation experiment, real-time gamma spectroscopy measurements were possible utilizing the NPI detector from PHDS. With this data, maps of the ^76/77^Kr distribution, as it flowed through the system and accumulated on the cold traps, were created. In addition to the real-time radiation maps, the gamma spectra made it possible to predict and model the mass transport of both ^76^Kr and ^77^Kr through the system based upon the production in the tank water, Henry’s law for the sparging of krypton into the gas phase, the flow of gas from the headspace to the traps for collection, and the decay of the ^76/77^Kr isotopes over the irradiation time. From this, the production rates of ^76^Kr and ^77^Kr were determined to be 2.7(1) × 10^–4^ nuclei of ^76^Kr and 1.18(6) × 10^–2^ nuclei of ^77^Kr formed per incident ^78^Kr ion. Looking forward to higher intensity irradiations at FRIB, it will be very beneficial to have the mass transport model of ^76/77^Kr through this system to apply the model to higher production rates.

After the online collection of radioactive ^76/77^Kr gas, utilizing differences in temperature, the gas was transferred between different vessels to generate samples of the daughter bromine isotopes. This ^76^Kr/^76^Br and ^77^Kr/^77^Br generator concept proved successful with the isolation of ^76^Br from ^77^Br. The ^76^Br and ^77^Br products were eluted from the vessels using water with an average efficiency of 77 ± 12%. Radionuclidically pure elutions of ^76^Br could be obtained by a separation based on different half-lives of mother/daughter radionuclides.

While this collection and generation method proved successful, the future of this work will involve more novel methods of noble gas capture including metal–organic frameworks (MOFs). MOFs can be designed to selectively capture certain gases over others and through this method, the efficiency of gas collection of the harvesting system could be improved.

Using the mass transport constants and production rates from this work, it is possible to extrapolate to the conditions expected at FRIB. When FRIB reaches full power, the anticipated steady-state radioactivities are around 50 GBq for ^76^Kr and 200 GBq for ^77^Kr while the ^78^Kr primary beam is in use. If harvested as described, around 5 GBq ^76^Kr will be available for extraction each day, which will be accompanied by 3 GBq of ^77^Kr. With the minor modification of adding membrane contactors to the harvesting system (in order to increase the degassing rate kinetics), the harvestable portion should exceed 25 GBq/day for ^76^Kr which will be accompanied by 50 GBq ^77^Kr . Although the ^78^Kr beam will only be run for about 10 days per year, the same techniques (with some modification) can be employed to capture Kr, Xe, and Rn isotopes during other irradiations. Since the majority of the scientific program at FRIB will involve a ^238^U primary beam, the major impact of gas phase harvesting will likely come from applying the techniques described here to collecting ^211^Rn for ^211^At generation. ^211^At is one of the few viable radionuclides for targeted alpha therapy, and increased access to it through isotope harvesting at FRIB will facilitate translational research which is currently supply-limited.

## Supplementary Information

Supplementary Information.

## References

[1] Gade A, Sherrill BM (2016). NSCL and FRIB at Michigan State University: nuclear science at the limits of stability. Phys. Scr..

[2] Abel EP, Avilov M, Ayres V, Birnbaum E, Bollen G, Bonito G, Bredeweg T, Clause H, Couture A, DeVore J, Dietrich M, Ellison P, Engle J, Ferrieri R, Fitzsimmons J, Friedman M, Georgobiani D, Graves S, Greene J, Lapi S, Loveless CS, Mastren T, Martinez-Gomez C, McGuinness S, Mittig W, Morrissey D, Peaslee G, Pellemoine F, Robertson JD, Scielzo N, Scott M, Severin G, Shaughnessy D, Shusterman J, Singh J, Stoyer M, Sutherlin L, Visser A, Wilkinson J (2019). Isotope harvesting at FRIB: additional opportunities for scientific discovery. J. Phys. G Nucl. Part. Phys..

[3] Domnanich KA, Abel EP, Clause HK, Kalman C, Walker W, Severin GW (2020). An isotope harvesting beam blocker for the national superconducting cyclotron laboratory. Nucl. Instruments Methods Phys. Res. Sect. A Accel. Spectrometers, Detect. Assoc. Equip..

[4] Abel EP, Domnanich K, Kalman C, Walker W, Engle JW, Barnhart TE, Severin G (2020). Durability test of a flowing-water target for isotope harvesting. Nuclear Inst. Methods Phys. Res. B..

[5] Abel EP, Clause HK, Severin GW (2020). Radiolysis and radionuclide production in a flowing-water target during fast ^40^Ca^20+^ irradiation. Appl. Radiat. Isot..

[6] Abel EP, Domnanich K, Clause HK, Kalman C, Walker W, Shusterman JA, Greene J, Gott M, Severin GW (2020). Production, collection, and purification of 47Ca for the generation of 47Sc through isotope harvesting at the national superconducting cyclotron laboratory. ACS Omega.

[7] Domnanich KA, Vyas CK, Abel EP, Kalman C, Walker W, Severin GW (2020). Harvesting ^62^Zn from an aqueous cocktail at the NSCL. New J. Chem..

[8] Mason NS, Mathis CA, Valk PE, Bailey DL, Townsend DW, Maisy MN (2003). Radiohalogens for PET imaging. Positron emission tomography: basic science and clinical practice.

[9] Glaser M, Luthra SK, Brady F (2003). Applications of positron-emitting halogens in PET oncology (Review). Int. J. Oncol..

[10] Stepanek J, Larsson B, Weinreich R (1996). Auger-electron spectra of radionuclides for therapy and diagnostics. Acta Oncol..

[11] Singh B (1995). Nuclear data sheets update for A=76. Nucl. Data Sheets.

[12] Singh B, Nica N (2012). Nuclear data sheets for A=77. Nucl. Data Sheets.

[13] Wilbur DS, Adam M (2019). Radiobromine and radioiodine for medical applications. Radiochim. Acta..

[14] Hassan HE, El-Azony KM, Azzam A, Qaim SM (2017). Investigation of selenium compounds as targets for ^76,77^Br production using protons of energies up to 34 MeV. Radiochim. Acta..

[15] Ellison PA, Olson AP, Barnhart TE, Hoffman SLV, Reilly SW, Makvandi M, Bartels JL, Murali D, DeJesus OT, Lapi SE, Nickles RJ, Mach RH, Engle JW (2020). Improved production of ^76^Br, ^77^Br, and ^80m^Br via CoSe cyclotron targets and vertical dry distillation. Nucl. Med. Biol..

[16] Tolmachev V, Lovqvist A, Einarsson L, Schultz J, Lundqvist H (1998). Production of ^76^Br by a low-energy cyclotron. Appl. Rad. Isotopes..

[17] De Jong D, Kooiman H, Veenboer JT (1979). ^76^Br and ^77^Br from decay of cyclotron produced ^76^Kr and ^77^Kr. Int. J. Appl. Radiat. Isot..

[18] De Jong D, Bakker CNM, Van Halteren BW, Kaspersen FM, Kooiman H (1982). Excitation labeling of simple organic molecules with ^76^Br and ^77^Br. Int. J. Appl. Radiat. Isot..

[19] McGuiness SR, Wilkinson JT, Peaslee GF (2021). Heavy-ion production of ^77^Br and ^76^Br; *Sci*. Rep..

[20] Cruz G (2013). Boric Acid in Kjeldahl Analysis. J. Chem. Educ..

[21] Freeman BS (2014). Chapter 17: Absorption of Carbon Dioxide.

[22] De Jong, D. Brinkman, G. A., Van Halteren, B. W. Inorganic ^76,77^Br containing products from the decay of ^76,77^Kr. *Int. J. Appl. Radiat Isot.* **37**(7), 621–622 (1986)

