# Supplementary material for: Harvesting krypton isotopes from the off-gas of an irradiated water target to generate 76Br and 77Br
Source: Sci Rep. 2022 Jan 26;12:1433. doi: 10.1038/s41598-022-05500-8 (PMC8792061; doi:10.1038/s41598-022-05500-8)
Supplement: Supplementary file 1 — Supplementary Information. [file 41598_2022_5500_MOESM1_ESM.docx]

# SUPPLEMENTAL MATERIAL

**Harvesting Krypton Isotopes from the Off-Gas of an Irradiated Water Target to Generate ^76^Br and ^77^Br**

Hannah K. Clause, Katharina A. Domnanich, Chloe Kleinfeldt, Morgan Kalman, Wesley Walker, Chirag Vyas, E. Paige Abel, Gregory W. Severin

## Mass transport modeling:

Mass transport of Kr isotopes through the isotope harvesting system is dependent upon the instantaneous beam current, the rate of exchange between ^76/77^Kr dissolved in the water tank and gaseous ^76/77^Kr in the headspace, the flow rate of the sparging gas from the tank to the traps, and the decay of the produced gaseous ^76/77^Kr isotopes over the irradiation time.

The total activity of ^76/77^Kr gas in the harvesting system is described by:

$$\begin{aligned} dA_{total}=dt \left[ c_{1}I\left( t \right)- \lambda A_{total} \right]\#1 \end{aligned}$$

where *A_total_* is the total activity of ^76/77^Kr at a given time, *t*; *c_1_* is a production factor representing the production of the ^76/77^Kr isotope per incident charge; *I(t)* is the beam current measurement at a specific time, *t*; and $\lambda$ is the decay constant of the respective ^76/77^Kr isotope.

The total activity was separated into activity in different regions. The activity in the water tank, *A_tank_*, the tank headspace, *A_head_*, the cold traps, *A_trap_*, and the gas capture bags, *A_bag_* with mass balance being met through:

$$\begin{aligned} A_{total} = A_{tank} + A_{head} + A_{trap}+A_{bag} ,\#2 \end{aligned}$$

and the associated differential equation:

$$\begin{aligned} {dA}_{total} = {dA}_{tank} + {dA}_{head} + {dA}_{trap}+{dA}_{bag} .\#3 \end{aligned}$$

Due to the rapid flow rate of the water between the tank and the target, the activity in the target and transfer lines was considered to be part of the tank in the model. Combining this with the exchange kinetics of dissolved gas between the liquid phase and the headspace gas phase, *k_1_* and *k_2_*, and the radioactive decay gives a differential equation for *A_tank_*:

$$\begin{aligned} {dA}_{tank}= dt \left[ c_{1}I\left( t \right)- \lambda A_{tank}- k_{1}A_{tank}+ k_{2}A_{head} \right] \#4 \end{aligned}$$

where *k_1_* and *k_2_* are fixed to each other by Henry’s law coefficient, *k_H_*, the water volume in the tank, *V_tank_*, the headspace gas volume *V_head_*, the temperature *T*, and the ideal gas constant *R* through:

$$\begin{aligned} k_{2}=k_{1}k_{H}\frac{V_{tank}}{V_{head}}RT \#5 \end{aligned}$$

Similarly, differential expressions for *A_head_* and *A_trap_* are found by including a gas flow rate, *f*, term:

$$\begin{aligned} {dA}_{head}= dt \left[ - \lambda A_{head}+ k_{1}A_{tank}- k_{2}A_{head}-{\frac{f}{V_{head}}A}_{head} \right] \#6 \end{aligned}$$

and

$$\begin{aligned} {dA}_{trap}= dt \left[ - \lambda A_{trap}+{\frac{f}{V_{head}}A}_{head}-{\delta A}_{trap} \right]\#7 \end{aligned}$$

where $\delta$ is a fractional trap inefficiency per unit time, representing activity transfer out of the traps and into the gas capture bags according to:

$$\begin{aligned} {dA}_{bag}= dt \left[ - \lambda A_{bag}+{\delta A}_{trap} \right] \#8 \end{aligned}$$

The entire model is graphically represented as a box diagram shown in **Figure S1**. Due to the non-functional form of the beam-current’s time dependence, the differential equations were numerically integrated with time steps, *dt*, not exceeding 10 s, to yield time-dependent representations of the activities in each ROI. Using the NPI, 13 gamma measurements were made throughout the irradiation. The relative time in seconds and scaled activity results for ^77^Kr can be seen in Table S1. These measurements were used for the comparison between the predicted mass transport model and the real-time data collected during the irradiation in Figures 6A and 6B in the main text.

The NPI data (denoted here as $\bar{A}_{tank+head}$ and $\bar{A}_{trap}$) and the activity measurements obtained with the BEGe detector were used to fit, through chi-squared minimization, the constants *c_1_, k_1_, δ* and separate scaling constants for the tank-plus-headspace ROI, *α_1_*, and the cold trap ROI, *α_2_*, that accounted for the differing gamma-ray attenuation between the ROIs in the NPI detector.

The fit equations were:

$$\begin{aligned} \bar{A}_{tank+head}= \frac{\alpha_{1}}{t_{off}-t_{on}}\int_{t_{on}}^{t_{off}} \left[ A_{tank}+A_{head} \right] dt \#8 \end{aligned}$$

and

$$\begin{aligned} \bar{A}_{trap}= \frac{\alpha_{2}}{t_{off}-t_{on}}\int_{t_{on}}^{t_{off}} A_{trap} dt\#9 \end{aligned}$$

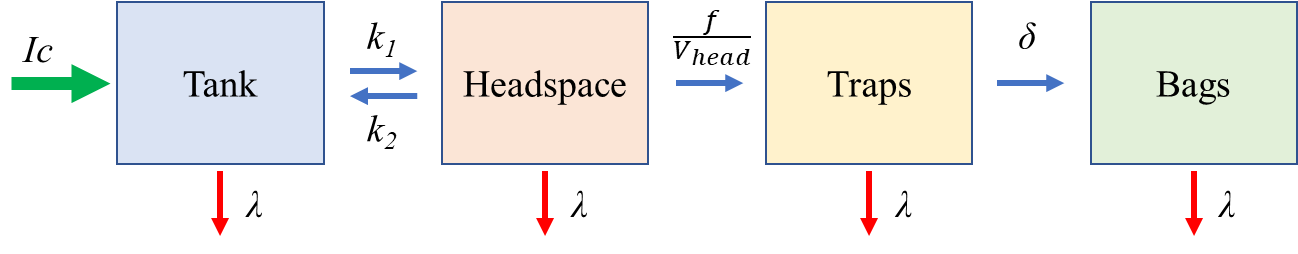


**Figure S1:** Box diagram representing the mass transport model of krypton gas as it travelled through the system and gas line.

**Table S1:** Quantifications obtained from the NPI. Note: These values were calibrated against the data obtained with the BE2022 detector. Therefore, the activities presented here are dependent upon the mass transport model through the linear scaling factor for each ROI, *α_1_* and *α_2_*.

| NPI  Time Scale (s) | Tank + Headspace Activity (MBq) | Tank + Headspace  Uncertainty | Trap  Activity (MBq) | Trap  Uncertainty |
| --- | --- | --- | --- | --- |
| 0 | 0.61 | 0.09 | 0.21 | 0.02 |
| 5407 | 2.96 | 0.20 | 0.57 | 0.03 |
| 5578 | 2.74 | 0.11 | 0.58 | 0.02 |
| 20204 | 2.57 | 0.21 | 1.22 | 0.07 |
| 21287 | 2.23 | 0.11 | 1.14 | 0.06 |
| 22155 | 2.12 | 0.17 | 1.05 | 0.01 |
| 22791 | 1.91 | 0.16 | 1.07 | 0.03 |
|  |  |  |  |  |

## Gamma Spectra and Quantifications:

Figure S2 shows the activity of both ^76/77^Kr and ^76/77^Br radioisotopes in the molecular sieve trap before and after the transfer into trap 2 for combination. These gamma spectra are depicted using the square root of counts to more easily observe all relevant peaks as opposed to the most intense peaks dominating the spectra. After transfer from the molecular sieve trap into trap 2, the activity of ^76^Kr and ^77^Kr decreased significantly, demonstrating the transfer of the gas out of the trap.


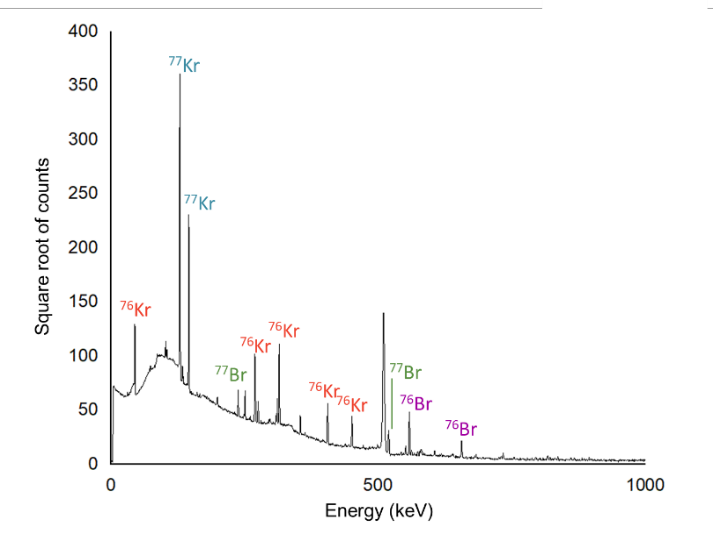

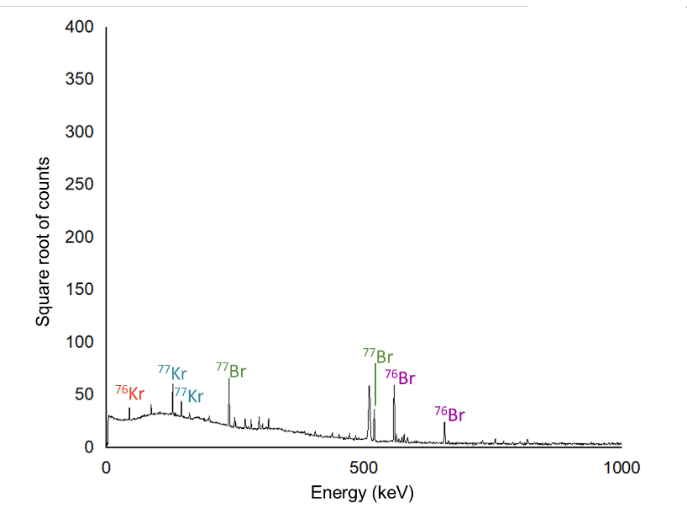


**Figure S2:** Gamma spectroscopy measurements of the molecular sieve trap before (left) and after (right) the transfer of krypton gases to stainless steel trap 2. The successful movement of krypton gas due to changes in temperature is demonstrated by the decrease in counts of activity of ^76^Kr and ^77^Kr.

Figure S3 shows the activity present in trap 3 after the transfer of gas to it from trap 2. This spectrum shows the isolation of ^76^Kr after most of the ^77^Kr decayed in order to generate only ^76^Br in trap 3.


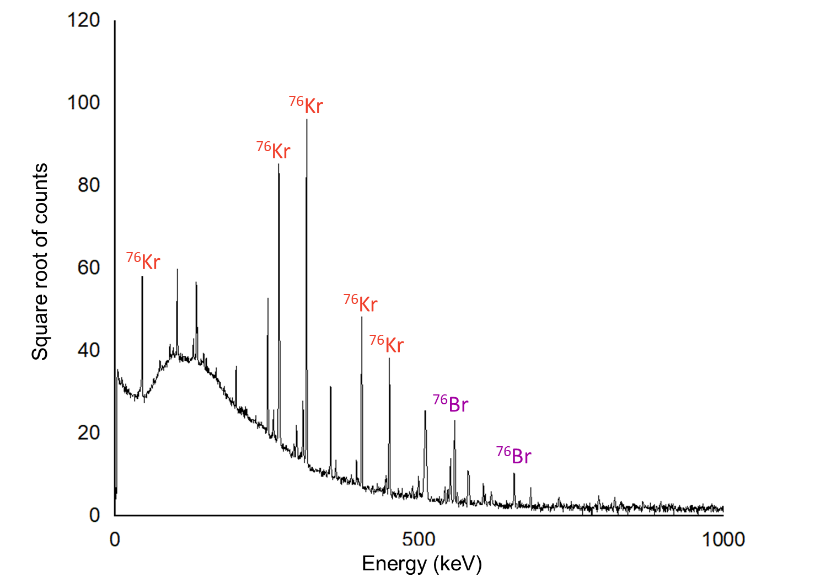


**Figure S3:** Gamma spectroscopy measurements of stainless steel trap 3 after the transfer of krypton gases from stainless steel trap 2. The successful movement of krypton gas due to changes in temperature is demonstrated by the resulting activity counts measured in trap 3 after the transfer. This also demonstrates the successful isolation of ^76^Kr in order to generate ^76^Br.

Figure S4 shows the activities of ^76^Kr and ^76^Br in trap 3 before the transfer out of it and the resulting activities in trap 4 after the transfer into it. This demonstrates the successful movement of ^76^Kr.


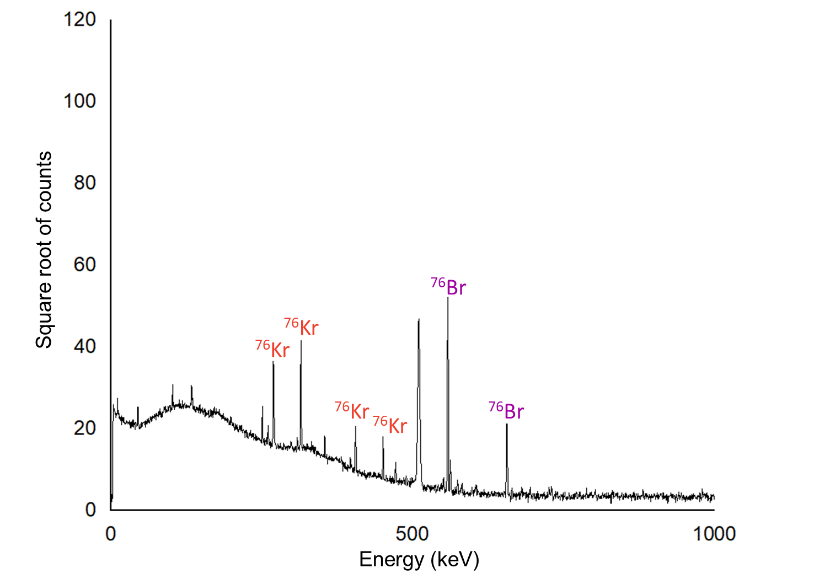

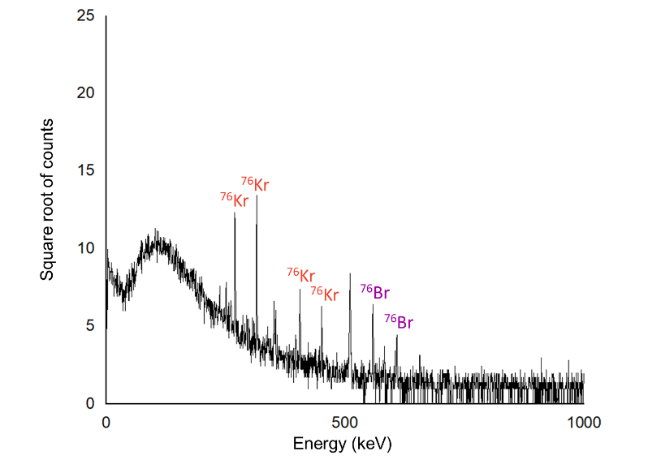


**Figure S4:** Gamma spectroscopy measurements of stainless steel trap 3 (left) before the transfer of ^76^Kr gas to stainless steel trap 4 (right). The successful movement of krypton gas due to changes in temperature is demonstrated by the presence of ^76^Kr gas in stainless steel trap 4.

The entire rinse procedure for trap 1 is demonstrated in Figure S5, where the activity before rinsing, the activity present in elution 1, the activity present in elution 2, and the activity in the trap after rinsing are shown.


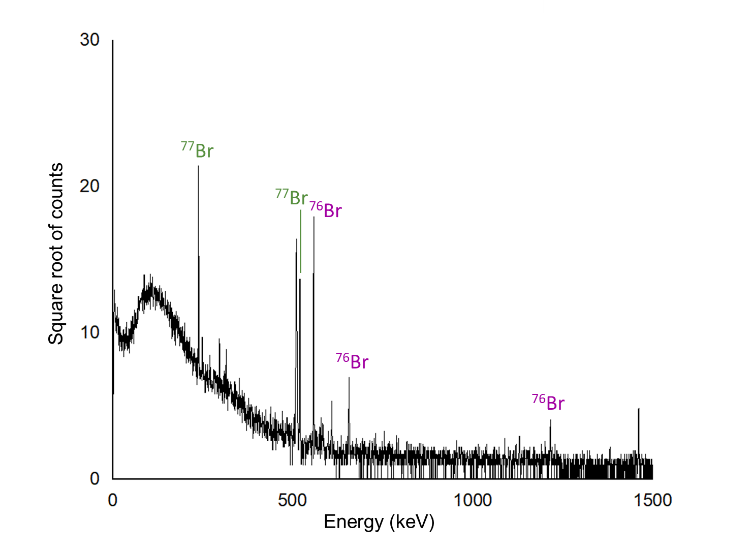

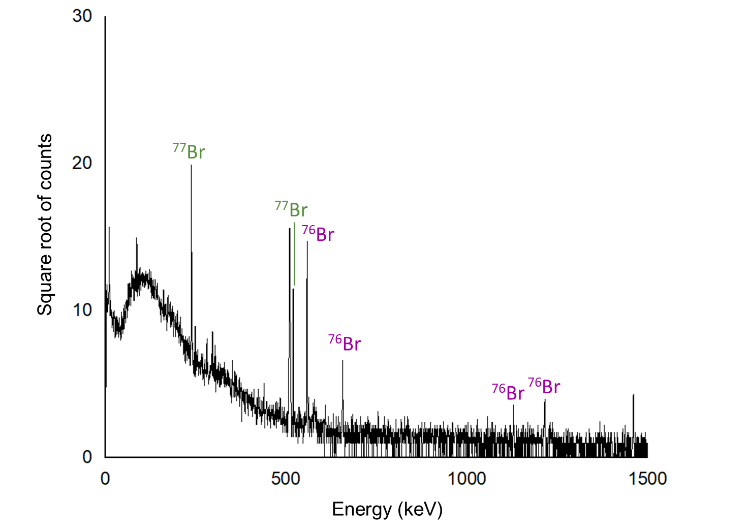


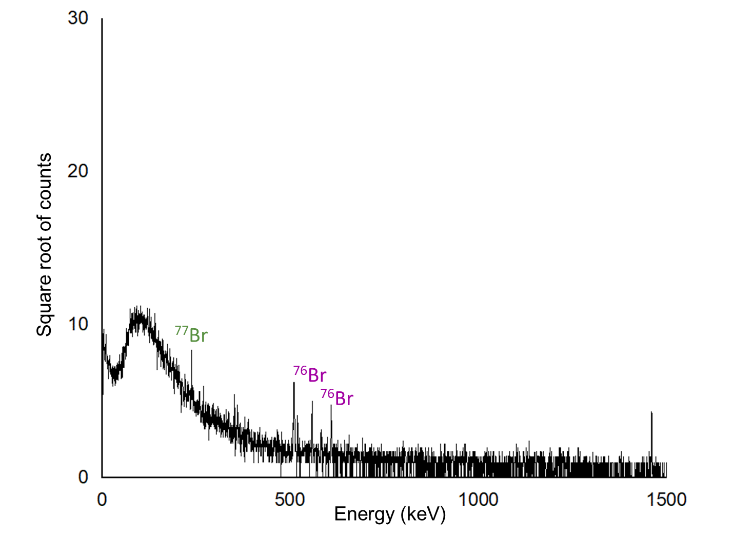

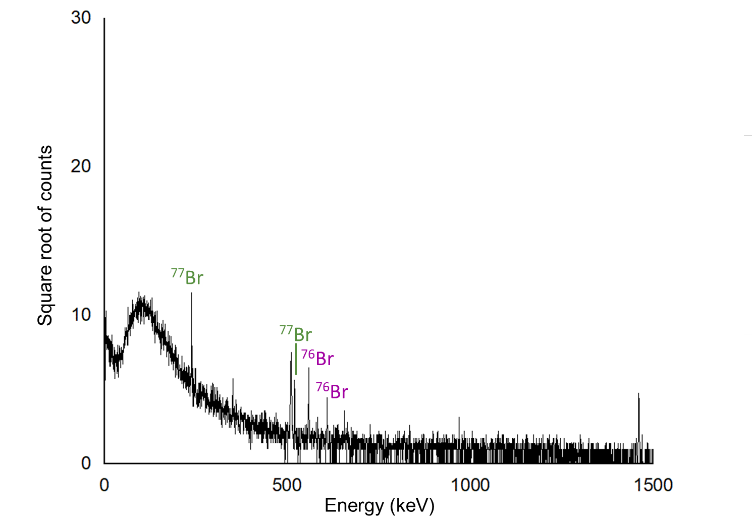


**Figure S5:** Trap 1 before elution, the eluent from wash 1, the eluent from wash 2, and the trap after washing.

The total activities of ^76/77^Br present in each trap prior to elution and present in each eluent step is shown in Table S2. The total amount of ^76/77^Br which was rinsed was calculated in order to determine the elution efficiency of each trial.

**Table S2:** Total bromine activity measurements (kBq) during elutions

|  | **Pre-elution** | **Elution 1** | **Elution 2** | **Elution 3** | **Total** | **Efficiency (%)** |
| --- | --- | --- | --- | --- | --- | --- |
| Trap 1 | 37 *(1)* | 29.6 *(7)* | 3.3 *(4)* | -- | 33 *(1)* | 89 |
| Trap 2 | 270 *(6)* | 181 *(4)* | 32.6 *(7)* | -- | 213 *(4)* | 79 |
| Trap 3 | 362 *(8)* | 170 *(4)* | 42 *(1)* | 18.5 *(7)* | 231 *(4)* | 64 |
